# Meta-Analysis: Hemodynamic Responses to Sub-anesthetic Doses of Ketamine in Patients With Psychiatric Disorders

**DOI:** 10.3389/fpsyt.2021.549080

**Published:** 2021-03-24

**Authors:** Jay Vankawala, Garrett Naples, Victor J. Avila-Quintero, Karina L. Ramírez, José M. Flores, Michael H. Bloch, Jennifer B. Dwyer

**Affiliations:** ^1^Yale Child Study Center, Yale University School of Medicine, New Haven, CT, United States; ^2^Yale Department of Psychiatry, Yale University School of Medicine, New Haven, CT, United States; ^3^Yale Department of Radiology and Biomedical Imaging, Yale University School of Medicine, New Haven, CT, United States

**Keywords:** ketamine, meta-analysis, hemodynamics, psychiatric disorders, blood pressure, heart rate

## Abstract

Ketamine, a medication traditionally used as an anesthetic, has increasingly been recognized as an effective treatment for psychiatric disorders. At sub-anesthetic doses (defined here as ≤ 0.5 mg/kg), ketamine treatment has been studied in patients with treatment-resistant depression (TRD), obsessive-compulsive disorder (OCD), post-traumatic stress disorder (PTSD), and social anxiety disorder (SAD). Transient increases in hemodynamic activity have been reported during and after ketamine treatment, which may be desirable properties in some anesthesia settings, but are generally undesirable in psychiatric settings. While ketamine doses used in psychiatry are lower than those used in anesthesia, there are published instances of early termination of psychiatric ketamine infusions due to elevations in blood pressure and heart rate. No unifying study has been conducted to examine the impact of sub-anesthetic ketamine doses on hemodynamic parameters [systolic blood pressure (SBP), diastolic blood pressure (DBP), and heart rate (HR)] in psychiatric populations and to evaluate these changes across adult age groups. Here, data from 15 articles comprising a total *N* = 2,252 ketamine or esketamine treatments in adult participants were used to conduct a meta-analysis of treatment-induced hemodynamic changes. Ketamine/esketamine produced modest but significant increases in the variables of interest with an average SBP increase of 12.61 mm Hg (95% CI 10.40–14.82 mm Hg, *z* = 11.18, *p* < 0.0001), average DBP increase of 8.49 mm Hg (95% CI 6.89–10.09 mmHg, *z* = 10.41, *p* < 0.0001), and average heart rate increase of 4.09 beats per minute (95% CI 0.55–7.63 BPM), *z* = 2.27, *p* = 0.0235). Stratified subgroup analysis indicated no significant differences between ketamine and esketamine effects on blood pressure. Further analysis indicated that there was no significant effect of age on ketamine-induced changes in SBP, DBP, and HR. Taken together these data show that sub-anesthetic ketamine and esketamine induce small but significant increases in hemodynamic parameters that are transient in nature in adult psychiatric populations. While these data are reassuring, it is important for each treatment case to fully explore potential cardiovascular risks prior to initiating treatment.

## Introduction

Major depressive disorder (MDD) is the most prevalent of all psychiatric disorders, with 17% of adults in the United States having a lifetime history of MDD (1). MDD is associated with significant costs, ranking as the second leading cause of years lived with disability globally (2), and presenting as a potent risk factor for suicide, now the second leading cause of death in young adults (3). More recent data has suggested that the worldwide incidence and burden of MDD has greatly increased in recent years with the number of incident cases worldwide increasing nearly 50% between 1990 and 2017 (4). Moderate MDD can be effectively treated with antidepressant monotherapy, psychotherapy, or a combination, and approaches to severe MDD include augmentation of antidepressants with antipsychotics or lithium, and interventional approaches (e.g., electroconvulsive therapy) (5). However, symptom resistance to treatment is common in clinical practice, as only one third of patients remit after a first antidepressant trial, and only two-thirds will achieve remission after four sequential treatments (6). Treatment resistant depression (TRD) presents a formidable public health challenge (7), and the search for novel therapeutics for this group is a top research priority.

Ketamine is a non-competitive antagonist of the N-methyl-D-aspartate (NMDA) receptor, one of the major glutamate receptor subtypes in the brain (8). While ketamine was originally purposed as a dissociative anesthetic (9), an increasingly large body of work demonstrates that sub-anesthetic doses of ketamine produce rapid antidepressant and anti-suicidal effects in adults with TRD (10–12). Psychiatric benefits have now been assessed across a variety of mental health disorders, alleviating symptoms of obsessive-compulsive disorder (OCD) (13), anxiety in patients with social anxiety disorder (SAD) (14), and PTSD symptoms (15, 16).

Ketamine differs from many anesthetics in that it increases cardiovascular activity and has a high threshold for respiratory depression (17). Other commonly used anesthetics, such as propofol and midazolam, show the opposite effects, with depressed hemodynamic readings during sedation (18). Consistent with the anesthesia literature, studies of both racemic ketamine (19) and the S-enantiomer, esketamine (20), show transient increases in hemodynamic responses. While elevated hemodynamic activity can be an advantage of ketamine compared to other agents in select patient populations during anesthesia, these effects are largely considered undesirable in the psychiatric setting. Indeed, some psychiatric studies report early termination of infusions due to blood pressure elevations that were unresponsive to antihypertensive treatment (21). Despite the importance of understanding potential adverse events, no unifying meta-analysis has been conducted to identify the hemodynamic changes caused by sub-anesthetic ketamine treatment across psychiatric populations. Quantifying these hemodynamic effects in psychiatric populations will allow a more comprehensive understanding of risk, aid in patient selection, and inform appropriate monitoring and safety protocols. Furthermore, as the use of ketamine and esketamine for psychiatric purposes begins to expand to older and younger patient populations (22–24), evaluating age-related risks for hemodynamic adverse effects is important. Thus, the goal of the current study is to appraise and meta-analyze the hemodynamic data (blood pressure (BP) and heart rate (HR) of existing studies of ketamine and esketamine treatment of adults with psychiatric disorders (e.g., TRD, OCD, PTSD, and SAD).

## Methods

Two authors (JV, GN) searched the electronic database of PubMed on July 29th, 2019 for relevant studies using the search: ((“Esketamine” [Supplementary Concept] OR “Esketamine” [All Fields] OR “ketamine” [All Fields] OR “ketamine” [MeSH Terms]) AND (“mental disorders” [MeSH Terms] OR (“mental” [All Fields] AND “disorders” [All Fields]) OR “mental disorders” [All Fields])) AND “clinical trial” [Filter].

The titles and abstracts of the studies obtained through the search were examined by two authors (JV, GN) in order to determine article inclusion. Discrepancies were addressed by the authors through discussion and conversation with the senior author (JBD). Studies that met eligibility for the meta-analysis passed the following criteria: (1) examining ketamine or esketamine treatment in adults (age > 18 years old) with psychiatric disorders and (2) clinical trials. Articles were excluded based on the following criteria: (1) No hemodynamic data or insufficient hemodynamic data, (2) Non-subanesthetic doses (>0.5 mg/kg), or (3) Ketamine or esketamine paired acutely with another drug or intervention (e.g., ECT). A study found outside of the initial literature search that passed all criteria (25) was also included in the final analysis. An additional four studies using intranasal esketamine (*N* = 1,305) were identified from the FDA publication of Advisory Committee Briefing Materials.

Data collected from each article included publication year, drug (ketamine or esketamine), mode of delivery (e.g., intravenous infusion, intranasal, oral, subcutaneous), dosage, sample size, baseline systolic blood pressure (SBP) (mean and standard deviation), maximum change in SBP (mean and standard deviation), baseline diastolic blood pressure (DBP) (mean and standard deviation), maximum change in DBP (mean and standard deviation), baseline heart rate (HR) (mean and standard deviation), maximum change in HR (mean and standard deviation), and time point along the infusion when the maximum SBP, DBP, and HR was observed. In all analyses, the maximum SBP, DBP, or HR value after infusion or administration was compared to the corresponding measure at baseline (prior to drug administration).

Fixed and random effects meta-analyses were performed for SBP, DBP, and HR. Due to high heterogeneity, we present random effects estimates for SBP and DBP. Estimates of heterogeneity were not significant for HR; therefore, we present fixed effects estimates. All studies provided the mean participant age, allowing us to perform meta-regression of each of the 3 hemodynamic outcomes onto age. We conducted stratified subgroup analyses to examine whether ketamine and esketamine demonstrated similar effects on blood pressure. Publication bias was assessed statistically using Egger's test for small studies and graphically using Funnel plots. Data management and all statistical analyses were completed using STATA/IC v16 (StataCorp LLC).

## Results

**Figure 1:**
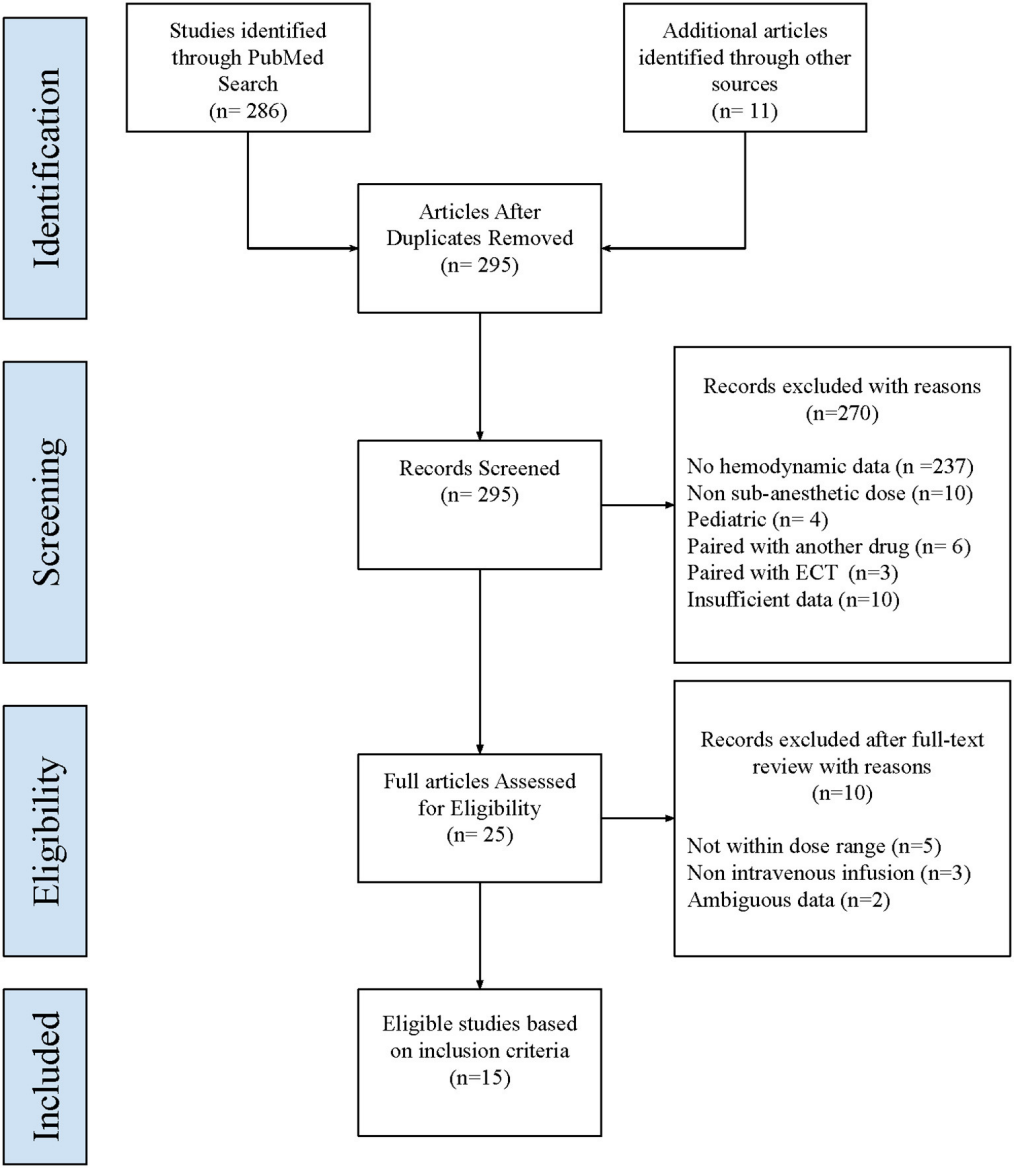
PRISMA diagram. This figure depicts the procedure for the selection of studies for meta-analysis.

Figure 1 is a PRISMA diagram that depicts the procedure for study selection. Our search yielded 286 citations during the initial systematic review. Further examination of these papers' full texts identified 11 studies involving *N* = 947 infusions in adult participants that were eligible for inclusion in this meta-analysis. An additional four studies using intranasal esketamine (*N* = 1,305) were identified from the FDA publication of Advisory Committee Briefing Materials. Table 1 lists the selected studies along with variables including the first author's name, year of publication, intervention drug and mode of administration (9 articles used intravenous ketamine, 1 used subcutaneous ketamine, 1 article used intranasal ketamine, and 4 used intranasal esketamine), dose used (9 studies used a single intravenous dose of 0.5 mg/kg, 1 intranasal ketamine study used a dose of 50 mg, 4 intranasal esketamine studies utilized a dose range of 28–84 mg, and a single study with subcutaneous administration used 3 different doses range from 0.1 to 0.5 mg/kg), sample size, average age of participants, indication for treatment (13 studies were designed for the indication of TRD, 1 for OCD, and 1 for SAD). The last variables collected were the outcomes studied in the meta-analysis (pre and post-SBP, DBP, and HR).

**Table 1:** Table of characteristics of the selected studies for the meta-analysis.

**References**	**Study design**	**Intervention drug**	**Mode of administration**	**Dose**	**Sample size**	**Age: mean (SD)**	**Indication for treatment**	**Outcome studied**
Riva-Posse et al. (26)	Retrospective analysis of clinical use	Ketamine	Intravenous	0.5 mg/kg	684	56.69 (12.86)	TRD	SBP DBP
Grunebaum et al. (27)	RCT, midazolam controlled, parallel	Ketamine	Intravenous	0.5 mg/kg	74	38.4 (13.2)	TRD	SBP DBP
Taylor et al. (14)	RCT, saline controlled, crossover	Ketamine	Intravenous	0.5 mg/kg	18	30.78 (13.50)	SAD	SBP DBP HR
Su et al. (28)	RCT, saline controlled, parallel	Ketamine	Intravenous	0.2, 0.5 mg/kg	47	46.75 (11.65)	TRD	SBP DBP HR
George et al. (29)	RCT, midazolam-controlled, multiple-crossover	Ketamine	Subcutaneous	0.1, 0.2, 0.3, 0.4, and 0.5 mg/kg	9	65.6 (5.7)	TRD	HR
Grunebaum et al. (27)	RCT, midazolam-controlled, parallel	Ketamine	Intravenous	0.5 mg/kg	15	39 (10.2)	TRD	SBP DBP
Vande Voort et al. (30)	Open-label trial	Ketamine	Intravenous	0.5 mg/kg	12	45.8 (8.0)	TRD	SBP DBP
Lapidus et.al. (31)	RCT, saline-controlled, crossover	Ketamine	Intranasal	50 mg	18	48.0 (12.8)	TRD	SBP DBP HR
Murrough et al. (21)	RCT, midazolam-controlled, parallel	Ketamine	Intravenous	0.5 mg/kg	47	46.9 (12.8)	TRD	SBP DBP
Bloch et al. (25)	Open-label trial	Ketamine	Intravenous	0.5 mg/kg	4	34.2 (9.0)	OCD	SBP DBP HR
Krystal et al. (32)	RCT, saline-controlled, crossover	Ketamine	Intravenous	0.1, 0.5 mg/kg	19	23.7 (0.9)	TRD	HR
Fedgchin et al. (33)	RCT, saline-controlled, parallel	Esketamine	Intranasal	56, 84 mg	116	46.05 (11.14)	TRD	SBP DBP
Daly et al. (34)	RCT, saline-controlled, parallel	Esketamine	Intranasal	56–84 mg	346	44.9 (12.58)	TRD	SBP DBP
Ochs-Ross et al. (35)	RCT, saline-controlled, parallel	Esketamine	Intranasal	28–84 mg	72	70.6 (4.79)	TRD	SBP DBP
Wajs et al. (36)	Open-label study	Esketamine	Intranasal	28–84 mg	771	52.2 (13.69)	TRD	SBP DBP

**Figure 2:**
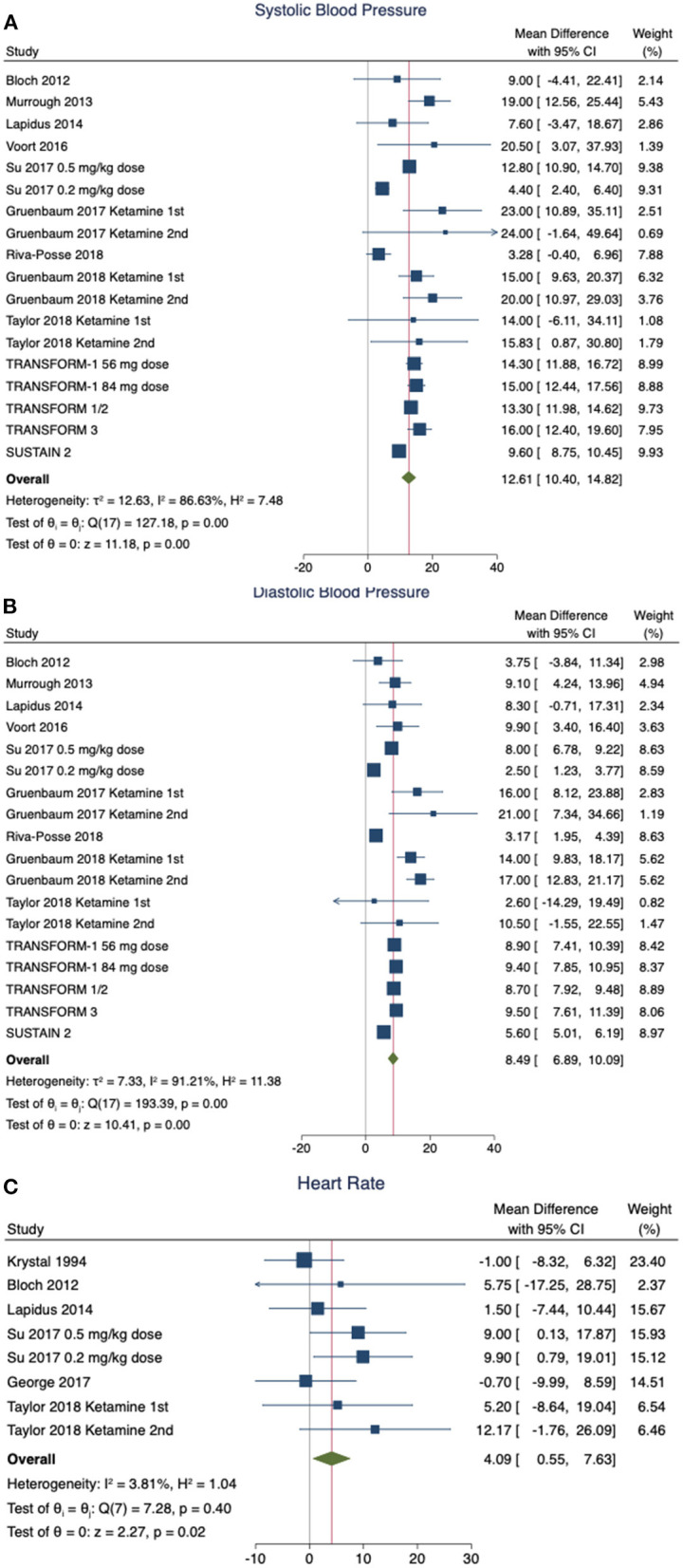
Forest plots. These plots depict pooled effects showing the mean difference in each hemodynamic parameter, and the heterogeneity for pre-post ketamine administration data. Mean and 95%CI are shown for systolic blood pressure (SBP) **(A)**, diastolic blood pressure (DBP) **(B)**, and HR **(C)**. Random effects models were used for **(A,B)**. A fixed effects model was used for **(C)** due to the low heterogeneity in the HR data.

Figure 2 shows forest plots estimating pooled effects and tests for heterogeneity for pre-post ketamine or esketamine administration differences for SBP, DBP, and HR using random effects models. Comparisons are between the pre-treatment baseline and the maximum value obtained at any timepoint after the start of medication administration. The random effect pooled estimate for pre-post SBP (Figure 2A) demonstrates an average increase of 12.61 mm Hg (95% CI 10.40–14.82 mm Hg, *z* = 11.18, *p* < 0.0001) with significant heterogeneity between studies (*I*^2^ = 86.63%, *Q* = 127.18, *p* < 0.0001). Likewise, for DBP (Figure 2B) there was an average increase of 8.49 mm Hg (95% CI 6.89–10.09 mmHg, *z* = 10.41, *p* < 0.0001) with significant heterogeneity between studies (*I*^2^ = 91.21%, *Q* = 193.39, *p* < 0.0001). Lastly, in terms of HR (Figure 2C), the pooled estimate was a mean increase in 4.09 beats per minute (BPM) (95% CI 0.55–7.63 BPM), *z* = 2.27, *p* = 0.0235) with non-significant heterogeneity between studies (*I*^2^ = 3.81%, *Q* = 7.28, *p* = 0.4006). Stratified subgroup analysis indicated that ketamine and esketamine demonstrated similar effects on SBP (Test of group differences *X*^2^ = 0.06, df = 1, *p* = 0.80) and DBP (Test of group differences *X*^2^ = 0.18, df = 1, *p* = 0.67). The four esketamine studies did not include heart rate data, and thus a subgroup analysis was not performed for heart rate.

**Figure 3:**
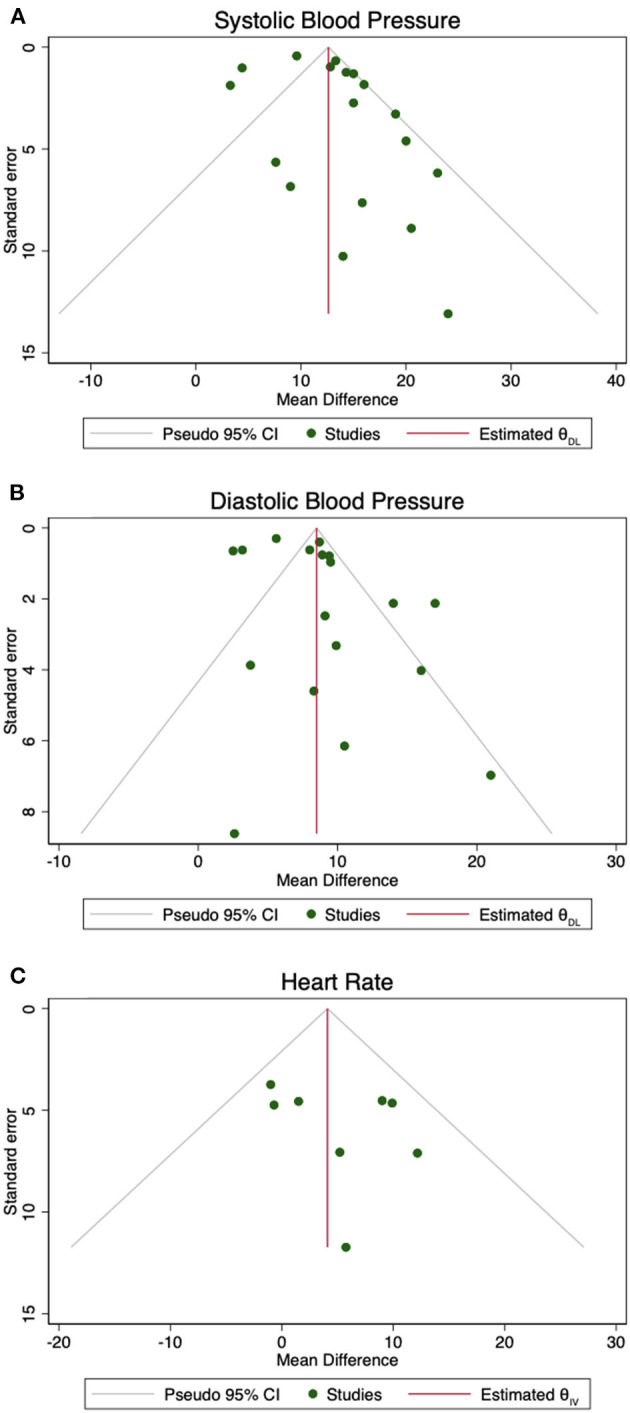
Funnel plots. These plots depict the standard error against the log mean difference in order to assess for publication bias. Symmetry in **(A)** SBP, **(B)** DBP, and **(C)** HR provides an assessment of publication bias, verified by the Egger's test.

Figure 3 presents funnel plots to assess publication bias. Although the Egger's test indicated publication bias for the DBP outcome (*p* = 0.0446), there was no significant evidence of publication bias for SBP (*p* = 0.0804) or HR (*p* = 0.3030). Graphically publication bias was evaluated by funnel plot symmetry.

### Meta-Regression of Hemodynamic Response to Ketamine Administration Across Average Adult Age Groups

**Figure 4:**
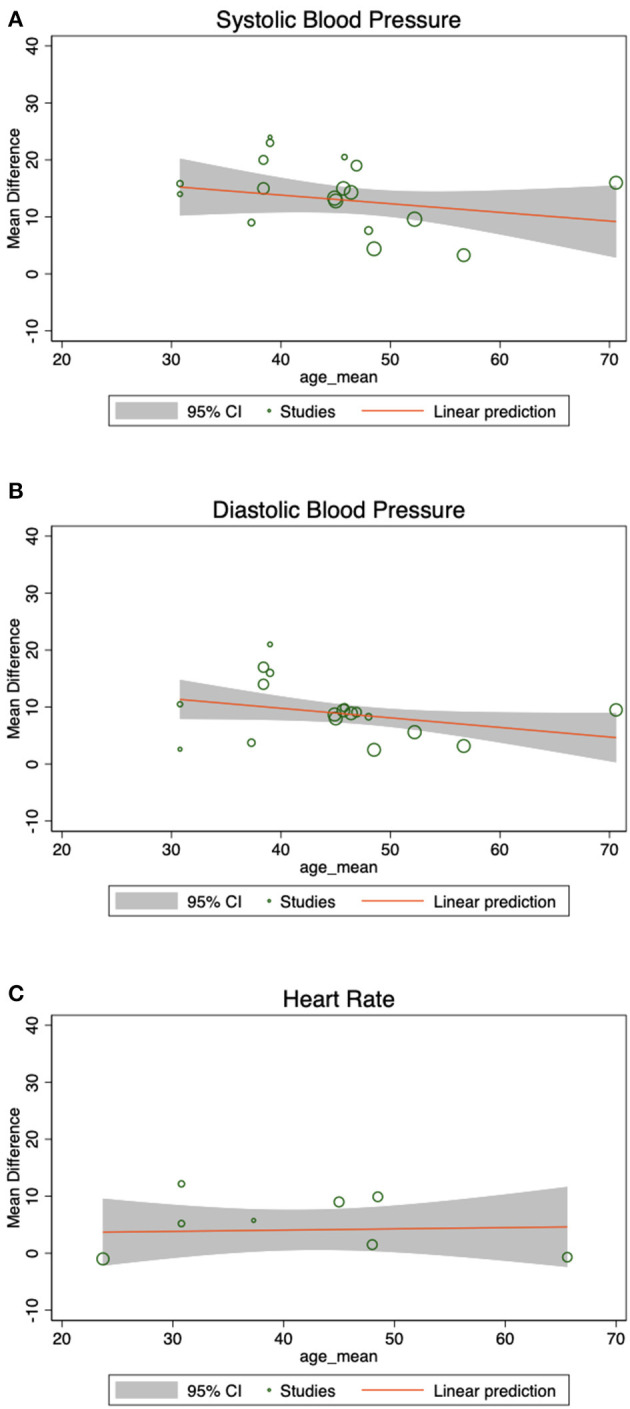
Meta-regression by age. These weighted scatter plots display hemodynamic data and its relationship with age, given by the meta-regression coefficient, showing that the association between mean age and **(A)** SBP, **(B)** DBP, and **(C)** HR was not statistically significant.

Meta-regression shows that there are no statistically significant effects of mean age on SBP (β = −0.15, 95% CI = −0.41 to 0.11, *z* = −1.14, *p* = 0.253), DBP (β = −0.17, 95% CI = −0.35 to 0.01, *z* = −1.84, *p* = 0.065), or HR (β = 0.02, 95% CI = −0.24 to 0.28, *z* = 0.17, *p* = 0.867). Figure 4 shows the weighted scatter plot of SBP, DBP, and HR and the relationship with age given by the meta-regression coefficient for the three hemodynamic outcomes of interest.

## Discussion

Here, we quantified the magnitude and variance of three important hemodynamic changes attributable to ketamine and esketamine administration in studies of psychiatric populations via meta-analysis. Ketamine/esketamine-induced blood pressure increases were statistically significant but overall modest, with an average post-administration maximum SBP of 132.48 mm Hg (SD = +/−10.19) and maximum DBP of 82.92 mm Hg (+/−5.80). These values meet the criteria for Stage 1 hypertension, defined as SBP values of 130–139 mm Hg or DBP of 80–89 mm Hg (37), but are far from meeting criteria for hypertensive urgency [SBP >180 mm Hg or DBP> 110 mm Hg (38)]. All studies reporting hemodynamic data included in this study concluded that the reported increases in blood pressure were transient and resolved soon after treatment cessation, although a single publication noted early infusion termination due to abnormally increased hemodynamic activity (21). The mean increases described here for participants with psychiatric disorders (12.61 mm Hg above baseline for SBP and 8.49 mm Hg above baseline for DBP) are similar to those reported in healthy controls [13 mm Hg above baseline for SBP and 13 mm Hg above baseline for DBP (39)], and there were no differences identified between ketamine and esketamine effects on blood pressure via stratified subgroup analysis. Lastly, the mean maximum HR was found to be 77.58 BPM (+/– 6.35) with a mean increase in HR from baseline to post-ketamine administration of 4.09 BPM, which falls within the normal range for adults (37). Thus, the meta-analyzed estimates of hemodynamic parameters in psychiatric participants undergoing ketamine or esketamine treatment were relatively small and stayed within the bounds of transient increases to Type I hypertension values. That said, reports of individual participants experiencing significant blood pressure elevations (21) highlight the need to determine factors associated with exaggerated hemodynamic responses.

In healthy subjects, predictors of enhanced ketamine-induced hypertension include higher baseline SBP, female gender, and those carrying the rs28386840 [T] allele of the norepinephrine transporter (39), which is associated with lower transporter expression and reduced norepinephrine reuptake capacity (40). Here we investigated the impact of age on ketamine/esketamine-induced hemodynamic changes. In the psychiatric population included in this analysis, meta-regression indicated that age did not significantly influences ketamine/esketamine-induced increases in SBP and DBP (Figure 4). One might expect enhanced risks for ketamine-induced hypertension in older adults, as age studies of cardiovascular function suggest faster SBP recovery times in young vs. older patients (41), and the incidence of essential hypertension and the risk for cardiovascular disease increases with age (42). However, the use of antihypertensive medication also increases with age (43) and standing antihypertensive medications could provide a buffer on ketamine-induced blood pressure increases. Unstable or untreated hypertension is also often an exclusion criterion in psychiatric ketamine studies, so the patients who may be most vulnerable to more severe blood pressure elevations are likely absent from the current analysis.

While the current study was unable to assess the role of medication effects, future studies should consider not only the role that anti-hypertensives may have in informing adverse event risk, but also the role of more traditional psychiatric medications that can have hemodynamic effects. Serotonin norepinephrine reuptake inhibitors (SNRIs), for example, block norepinephrine reuptake and dose-dependently raise blood pressure (44). Reduced norepinephrine reuptake may contribute to exaggerated ketamine-induced blood pressures increases in healthy participants (39), and thus, the impact of psychiatric medications that impact noradrenergic systems should be investigated. As ketamine is increasingly being studied and used clinically in broader patient populations with refractory mental illness (23, 45), influences of age, medication regimen, and medical co-morbidities on risk for cardiovascular adverse events should be revisited as more data become available.

Taken together, these data suggest that sub-anesthetic ketamine and esketamine cause increases in blood pressure and heart rate in adults with psychiatric disorders. Limitations of the current study include insufficient data to examine the influence of co-occurring medication regimens on ketamine-induced hemodynamic change. An additional limitation is the absence of PTSD studies that met inclusion criteria, as patients with PTSD may have additional risk factors for hypertension (46), which may or may not impact risk for ketamine-induced hemodynamic changes. While our data suggest that age may not significantly influence the degree of ketamine-induced hypertensive response, additional studies are needed to better define populations that may be at increased risk for exaggerated cardiovascular effects with ketamine treatment. While these data are reassuring at the group level, each individual case must carefully weigh all potential medical and psychiatric risks and benefits before proceeding with treatment.

## Data Availability Statement

The raw data supporting the conclusions of this article will be made available by the authors, without undue reservation.

## Author Contributions

JV conceived of the presented idea. VA-Q created a viable PubMed search string. JV, KR, and GN reviewed all articles and extracted all relevant data. VA-Q and JF analyzed extracted data. MB conducted the statistical analysis. JV, GN, VA-Q, and JF created figures and tables for the final publication. JD supervised all stages of the project. All authors discussed the results and contributed to the final manuscript.

## Conflict of Interest

JF receives financial support for his research from NIH (T32MH018268). MB receives research support from Therapix Biosciences, Neurocrine Biosciences, Janssen Pharmaceuticals and Biohaven Pharmaceuticals, but none provided support for the current manuscript. MB gratefully acknowledges additional research support from NIH. JD receives consulting income from Axsome Therapeutics and research support from the Klingenstein Third Generation Foundation, the AACAP Junior Investigator Award, the National Institutes of Mental Health, and the Brain and Behavior Foundation. The remaining authors declare that the research was conducted in the absence of any commercial or financial relationships that could be construed as a potential conflict of interest.
